# A Novel Assisted Oocyte Activation Method Improves Fertilization in Patients With Recurrent Fertilization Failure

**DOI:** 10.3389/fcell.2021.672081

**Published:** 2021-07-21

**Authors:** Meng Wang, Lixia Zhu, Chang Liu, Hui He, Cheng Wang, Chenxi Xing, Jinming Liu, Liu Yang, Qingsong Xi, Zhou Li, Lei Jin

**Affiliations:** ^1^Reproductive Medicine Center, Tongji Hospital, Tongji Medical College, Huazhong University of Science and Technology, Wuhan, China; ^2^Department of Oncology, Tongji Hospital, Tongji Medical College, Huazhong University of Science and Technology, Wuhan, China

**Keywords:** total fertilization failure, cycloheximide, ionomycin, fertilization, assisted oocyte activation

## Abstract

Total fertilization failure (TFF) occurs in 1–3% of total intracytoplasmic sperm injection (ICSI) cycles and can reoccur in subsequent cycles. Despite the high success rate with the application of assisted oocyte activation (AOA), there is still a small number of couples who cannot obtain fertilized eggs after conventional calcium (Ca^2+^) ionophores-based ICSI-AOA. Six couples experiencing repeated TFF or low fertilization (<10%) after ICSI and conventional ICSI-AOA were enrolled in this study. Compared with the regular ICSI group and the conventional ICSI-AOA group, the new AOA method, a combination of cycloheximide (CHX) and ionomycin, can significantly increase the fertilization rate from less than 10 up to approximately 50% in most cases. The normal distribution of sperm-related oocyte activation factor phospholipase C zeta (PLCζ1) in the sperms of the cases indicated the absence of an aberrant Ca^2+^ signaling activation. The results of the whole-embryo aneuploidies analysis indicated that oocytes receiving the novel AOA treatment had the potential to develop into blastocysts with normal karyotypes. Our data demonstrated that CHX combined with ionomycin was able to effectively improve the fertilization rate in the majority of patients suffering from TFF. This novel AOA method had a potential therapeutic effect on those couples experiencing TFF, even after conventional AOA, which may surmount the severe fertilization deficiencies in patients with a repeated low fertilization or TFF.

## Introduction

Assisted reproductive techniques (ART) offers infertile couples the possibility to conceive, and the advent of intracytoplasmic sperm injection (ICSI), a process involving the injection of a single spermatozoon into the cytoplasm of a mature oocyte, has allowed the achievement of pregnancy for couples suffering from severe male factor infertility, low fertilization rates after conventional *in vitro* fertilization (IVF), or unexplained infertility. Although at present, a fertilization rate of approximately 70% is observed during ICSI cycles with a clinical pregnancy rate of 45%, total fertilization failure (TFF) still occurs in 1–3% of the total ICSI cycles and can reoccur in subsequent cycles, even when adequate numbers of mature oocytes are available (Nasr-Esfahani et al., 2010). TFF is generally a physical misery and emotional inadmissibility for infertile couples. Therefore, it seems to be significantly important to explore the reasons and seek the solutions of TFF.

There is now a general consensus that oocyte activation deficiency should be mainly responsible for TFF following conventional ICSI (Vanden Meerschaut et al., 2014b). Several studies have shown that the majority of couples suffering from TFF can benefit from assisted oocyte activation (AOA) following ICSI (Sfontouris et al., 2015; Bonte et al., 2019), and in some centers, AOA is usually offered to couples experiencing TFF or a very low fertilization rate after being well informed. The well-established and most commonly described AOA protocols can be classified into three different strategies, i.e., mechanical, electrical, and chemical stimuli (Nasr-Esfahani et al., 2010). Calcium (Ca^2+^) ionophore, including ionomycin and A23187 (also known as calcimycin), is one of the most widely used AOA agents in the human ART process. It aims to raise Ca^2+^ artificially in oocyte cytoplasm for Ca^2+^ oscillations and can either promote Ca^2+^ influx from the extracellular medium by altering the cellular membrane permeability or target directly on the endoplasmic reticulum (ER) to release Ca^2+^ (Swann, 2018). Despite the high success rate with the application of AOA, there is still a small number of couples who cannot obtain fertilized eggs after conventional ICSI-AOA (Vanden Meerschaut et al., 2012).

It is generally accepted that oocyte activation in mammals is a complicated and spatial-temporal regulated process triggered by the entry of the sperm. Several minutes after sperm-oocyte plasma membrane interaction, intracellular Ca^2+^ oscillations flowing from the ER are induced by the release of a soluble sperm factor named phospholipase C zeta (PLCζ). A series of calcium-sensitive downstream pathways are then activated by these Ca^2+^ changes, further resulting in the inactivation of the maturation promoting factor (MPF), the block of which conversely contributes to the occurrence of the early events of oocyte activation, such as the restart of meiosis and the discharge of the second polar body (Kashir et al., 2013; Vanden Meerschaut et al., 2014b). Oocyte activation is a series of calcium-sensitive reactions followed by MPF inactivation, and conventional ICSI-AOA is applicable for the majority of TFF, which was mainly attributed to the lack of Ca^2+^ oscillations. Therefore, we conjecture that the failed conventional ICSI-AOA was caused by the abnormality of the downstream modulators after Ca^2+^ oscillations including MPF. The regular clinical strategy for those patients was donor insemination. However, with the rapid development of novel gene testing techniques, we tried to seek out the causes of TFF after ICSI-AOA and its possible treatment strategy.

The inactivation of MPF is considered as one of the most important links of oocyte activation as Ca^2+^ oscillations. Cycloheximide (CHX), a non-specific protein synthesis inhibitor, can inhibit the synthesis of cyclin B and cytostatic factor (CSF), keep MPF at a low level, and finally lead to oocyte parthenogenetic activation. Therefore, CHX was applied as an assistant agent on the parthenogenetic activation of mammals, like bovine, equine, and feline oocytes (Grabiec et al., 2007; Fernandes et al., 2014). In addition, some pieces of literatures have reported that ionomycin is a more specific Ca^2+^ ionophore with higher reported fertilization rates than A23187 (Nikiforaki et al., 2016). Hence, we assumed that CHX combined with ionomycin may be able to work as a novel treatment for those couples dealing with repeated TFF or low fertilization rates and without improvement with the conventional AOA. The present study aimed to put forward a novel activation protocol applied in a repeated complete or incomplete fertilization failure after ICSI-AOA.

## Materials and Methods

### Study Design and Participants

A total of six couples undergoing ART treatment in the Reproductive Medicine Center, Tongji Hospital, Huazhong University of Science and Technology between January 2018 and December 2019 were enrolled in the study. Those couples experienced repeated failed or low fertilization (<10%) after ICSI, and conventional ICSI-AOA (using Ca^2+^ ionophore only, such as ionomycin or A23187) (Vanden Meerschaut et al., 2012) did not work for these patients. All enrolled participants when signing a consent form were asked to undergo an oocyte activation test (OAT) after ICSI-AOA failure. OAT was an effective diagnostic test to help distinguish whether TFF should be attributed to sperm- or oocyte-related deficiencies (Sang et al., 2018). Generally, sperm from the TFF couples was injected into 6–10 *in vitro* maturation (IVM) MII oocytes donated by other identified normal IVF individuals. For the OAT analysis, if the fertilization rate was comparable to the normal fertilization rate of the IVM MII oocytes (about 50% or over), it indicated that the sperm from the TFF couples was normal, and the maternal factors should be responsible for TFF. If the fertilization rate was still less than 10% or zero, it demonstrated that the sperm from the TFF couples was abnormal. The OAT analysis for each couple was performed at least three times independently. This study was approved by the Ethics Committee of Tongji Hospital, Tongji Medical College, Huazhong University of Science and Technology (TJ-IRB20200722).

### Semen Analysis and Preparation

Fresh ejaculated semen was obtained by masturbation and collected on the oocyte retrieval day in sterile containers and kept for half an hour at 37∘C for liquefaction followed by concentration, total motility, viability, and morphology analyses under the light microscope. The semen analysis was based on the standard of the fifth edition of the WHO guidelines. A normal semen sample should be equipped with at least a concentration of 15 × 10^6^/ml, a total motility of 40%, and a normal morphology rate of 4% (Xin et al., 2020). Standard density-gradient centrifugation was performed for sperm selection as previously described (Huang et al., 2015). Briefly, 1.5 mL of 45% gradient media (Vitrolife, Gothenburg, Sweden) was added on the top of 1.5 mL of 90% gradient media. Then, up to 3 mL of the semen was layered on the gradient media and centrifuged at 200 *g* at room temperature for 20 min. The sperm pellet was isolated and washed with 3 mL of Sperm Washing Medium (Vitrolife, Gothenburg, Sweden) at 300 g for 6 min. Then, the sperm pellet was resuspended in 0.5 mL of Sperm Washing Medium and left at room temperature to allow for a swim-up for 30–60 min. The top 300 μL was collected for semen parameters analysis.

### Oocyte Retrieval and Fertilization

All participants underwent a controlled ovarian stimulation that was processed based on the previous study (Wang et al., 2021). Oocytes were retrieved by transvaginal ultrasound 36–38 h after HCG administration. For IVF patients, oocytes got fertilized 3–4 h after oocyte retrieval, and degranulation occurred 4 h after fertilization. If the second polar body had not been observed until 6 h after fertilization, early rescue ICSI was performed after the patients signed a consent form. For ICSI patients, cumulus-oocyte complexes were exposed to 80 U/L hyaluronidase (Irvine Scientific, United States) followed by mechanical pipetting for degranulation. Nude oocytes were further cultured for another 1–2 h before spermatozoon injection. Generally, pronucleus (PN) assessments were performed 17–18 h after fertilization.

### Conventional ICSI-AOA Procedure

Conventional ICSI-AOA procedure was performed as previously described (Capalbo et al., 2016). Briefly, freshly collected MII oocytes were exposed to pre-equilibrated Ca^2+^-ionophore A23187 (GM508 Cult-Active, Gynemed, Germany) for 15 min after the regular sperm injection.

### Novel ICSI-AOA Procedure

Novel ICSI-AOA test was an experimental test to explore effective methods to rescue fertilization failure. For paternal-factor-induced fertilization failure, injected oocytes were acquired from donated immature oocytes and *in vitro* mature oocytes from other IVF individuals. For maternal-factor-induced fertilization failure, oocytes were donated by the female patient with a written consent to research. Since ionomycin was reported to be a more specific Ca^2+^ ionophore with higher reported fertilization rates than A23187, in the novel ICSI-AOA test procedure, oocytes after ICSI were immediately transferred to the ionomycin (5 μM, MB7511, Meilunbio, Dalian, China) medium for 5 min of incubation before another 6 h interval in the CHX (10 μg/ml, a gift from Chinese Academy of Sciences) medium which was pre-equilibrated for at least 6 h. After AOA, oocytes were then transferred into the G1-plus medium (Vitrolife, Gothenburg, Sweden) for future culture after washing.

### Embryo Culture and Analyses

The whole fertilization and embryo development processes were recorded by time-lapse monitoring. Embryos were cultured in the G1-plus medium until D3 and in the G2-plus medium (Vitrolife, Gothenburg, Sweden) from D3 to D6. The three main morphological parameters of the cleavage stage embryos were as follows: (a) the number of blastomeres, (b) the percentage of fragmentation, and (c) the variation in blastomere symmetry. Blastocyst morphology evaluation was based on the Gardner scoring system. Those successfully fertilized and well-developed blastocysts acquired from novel AOA tests were further analyzed by whole-embryo aneuploidy analysis. An amplification method named multiple annealing- and looping-based amplification cycles (MALBAC) was used for whole-genome amplification of the embryos, followed by next-generation sequencing (NGS). MALBAC ensures uniform amplification of the original genomic DNA and therefore reduces amplification bias (Zong et al., 2012). Detailed protocol has been presented in a previous study (Zhu et al., 2021).

### Immunofluorescence Staining

The sperms from normal donors and the cases were stained following the standard immunofluorescences protocol (Grasa et al., 2008; Lee et al., 2014). Fresh washed sperm samples were spotted on slides pre-coated with 0.1% poly L-lysine (Sigma, St. Louis, MO, United States). Anti-PLCζ1 antibody (1:100, bs-5378R) was purchased from Bioss for indirect immunofluorescences and the secondary antibody was labeled with Cy3 (1:200, Servicebio, Wuhan, China). Fluorescein isothiocyanate-conjugated peanut agglutinin (FITC-PNA; Invitrogen, United States) was used for direct acrosome stain with Hoechst-33342 (Sigma, St. Louis, MO, United States) for nuclei. The location of PLCζ1 and the status of acrosomes, together with the position of the nuclei, were determined using a fluorescence microscope (Axio Observer A1; Carl Zeiss, Germany).

### Statistical Analyses

All data were analyzed using the Statistical Package for the Social Sciences (SPSS 22.0, IBM, Armonk, NY, United States). The data were presented as mean ± standard deviation (SD) for continuous variables and percentages for categorical variables. The chi-square test and Fisher’s exact test were performed for continuous variables. The Kruskal-Wallis non-parametric rank-sum test was performed for continuous variables. Two-tailed hypothesis tests were performed; a *p*-value < 0.05 was considered to be statistically significant.

## Results

### The Clinical Characteristics of the Participants

In total, six couples with a history of repeated TFF after ICSI and non-effective conventional ICSI-AOA were included in this study, and the details of the characteristics of the couples are presented in Table 1. The average age of all the females was 28.8 ± 1.9 years old. All these couples had never conceived before with the infertility duration ranging from 2 to 8 years, and most of them were diagnosed with unexplained infertility (five out of six). Although the numbers of the retrieved oocytes, ranging from 4 to 27, were at an average level or even considered in terms of the female age, only one oocyte or less were fertilized with an extremely low fertilization rate, which was considered as failed or low fertilization after ICSI (<10%). Early rescue ICSI was performed in some cases (Cases 4 and 5), thus, all these patients have experienced at least one-time ICSI (including early rescue ICSI or conventional ICSI) and ICSI-AOA. In addition, all the other five couples (Cases 1–5) were identified to suffer from paternal-factor-induced TFF, and Case 6 was identified as maternal-factor-induced TFF by the OAT analysis. Oligoasthenotspermia can be observed in Case 1, and Cases 4 and 5 were detected as teratozoospermia (Supplementary Table 1). The semen parameters of the other cases were within the normal range in terms of concentration, motility, and morphology.

**TABLE 1:** The clinical characteristics of the enrolled cases.

	**Age** **(female/male)**	**Type of infertility**	**Infertility** **Duration** **(year)**	**Causes of infertility**	**IVF/ICSI cycle**	**Stimulation protocol**	**No. of oocytes retrieved**	**No. of MII oocytes**	**No. of fertilized oocytes**	**TFF causes** ^1^
Case 1	27/30	Primary	2	oligoasthenot spermia	ICSI	agonist	19	15	1	Paternal
					ICSI-AOA	agonist	21	19	0	
Case 2	27/28	Primary	4	unexplained	IVF	agonist	11	10	0	Paternal
					ICSI	agonist	8	6	0	
					ICSI-AOA	luteal-phase stimulation	13	10	1	
Case 3	32/32	Primary	8	unexplained	IVF + ICSI	agonist	16	(7 + 8)^3^	0	Paternal
					ICSI-AOA	agonist	11	7	1	
Case 4	30/31	Primary	6	unexplained	RICSI	agonist	11	10	1	Paternal
					ICSI-AOA	agonist	7	7	0	
Case 5	30/33	Primary	5	unexplained	RICSI	agonist	5	5	0	Paternal
					ICSI	agonist	4	4	0	
					ICSI-AOA^2^			7	1	
Case 6	27/35	Primary	8	unexplained	IVF	agonist	15	13	0	Maternal
					ICSI + ICSI-AOA	Antagonist	27	(7 + 7 + 8)^4^	0	

*TFF, total fertilization failure. RICSI, rescue ICSI was performed 4–6 h after conventional IVF. ICSI-AOA, ICSI with conventional assisted oocyte activation using Ca^2+^-ionophore A23187. ^1^TFF causes were identified by oocyte activation test (OAT). ^2^The oocytes for ICSI-AOA were donated IVM oocytes. ^3^Seven oocytes for IVF and eight for ICSI. ^4^Seven oocytes for ICSI, seven for ICSI-AOA, and the other eight oocytes for cryopreservation.*

### Identification of PLCζ1 Expression in Patients With TFF

**FIGURE 1:**
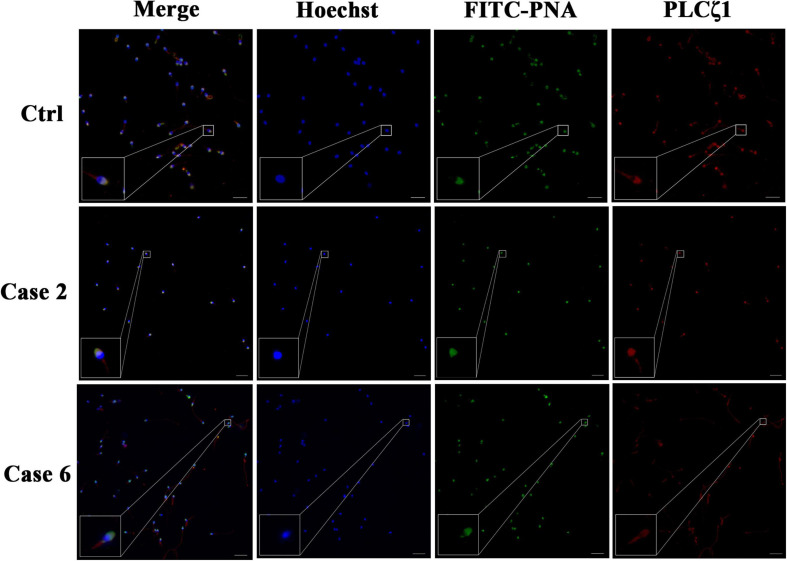
Detection of PLCζ1 expression in cases with TFF after conventional AOA. In the case of the normal male control, the signals of PLCζ1 (labeled by Cy3, red) overlapped with the outer acrosomal membrane marker PNA (labeled by FITC, green) almost completely. The localization and morphology of PLCζ1 and PNA in Cases 2 and 6 were in accordance with the normal control. The nuclei of the sperms were stained by Hoechst-33342 (blue). Scale bars = 50 μm.

Since conventional AOA by Ca^2+^ ionophore did not work with these patients and PLCζ1 was reported to be the main cause to induce Ca^2+^ oscillation, PLCζ1 expression was detected in the sperm of the male patients to explore the possible mechanism of TFF. Indirect immunofluorescence staining of PLCζ1 and direct acrosome stain were performed (Figure 1). In the normal sperm, the PLCζ1 signals overlapped with the signals of PNA, the marker of the outer acrosomal membrane of the sperm, almost completely. Similar results can be observed in the sperm of Case 2 (TFF caused by paternal factors) and Case 6 (TFF caused by maternal factors). Compared with the sperm of the normal control, the sperm of the couples experiencing TFF with failed conventional ICSI-AOA demonstrated a normal distribution of PLCζ1 and a normal morphology of acrosome. These observations indicated that PLCζ1 should not be attributed to all cases of TFF.

### The Validity Evaluation of the Novel AOA Method

As shown in Table 2, statistically significant differences can be observed among these three different groups in terms of the fertilization rate. Compared with the regular ICSI group and the conventional ICSI-AOA, the new oocyte activation method, a combination of CHX and ionomycin, can significantly increase the fertilization rate in most cases. Even for maternal-factor-related TFF in Case 6, where two frozen-thawed oocytes were donated by the patient to test the efficacy of the novel AOA, one egg was successfully fertilized.

**TABLE 2:** The comparison of the fertilization rates using different methods.

	**ICSI**	**Conventional ICSI-AOA**	**Novel ICSI-AOA**	***p*-value**
Case 1	1/15 (6.7%)_a_	0/19 (0.0%)_b_	7/16 (43.8%)_a b_	0.001*
Case 2	0/6 (0.0%)_a_	1/10 (10.0%)_b_	4/7 (57.1%)_a b_	0.033*
Case 3	0/8 (6.7%)	1/7 (14.3%)	1/8 (12.5%)	0.747
Case 4	1/10 (10.0%)	0/7 (0.0%)_a_	8/18 (44.4%)_a_	0.034*
Case 5	0/9 (0.0%)_a_	1/7 (14.3%)	7/12 (58.3%)_a_	0.010*
Case 6	0/7 (0.0%)	0/7 (0.0%)	1/2 (50.0%)^1^	0.056

Data were shown in number of fertilization oocytes/number of MII oocytes (percentage). Conventional ICSI-AOA: Ca^2+^-ionophore A23187 only. Novel ICSI-AOA: A combination of cycloheximide and ionomycin. ^1^The oocytes for novel ICSI-AOA were frozen-thawed oocytes from the affected female patient. _*a*__*b*_There were statistical differences between the groups labeled with the same letter. *p-values < 0.05 were considered to be statistically significant.

**FIGURE 2:**
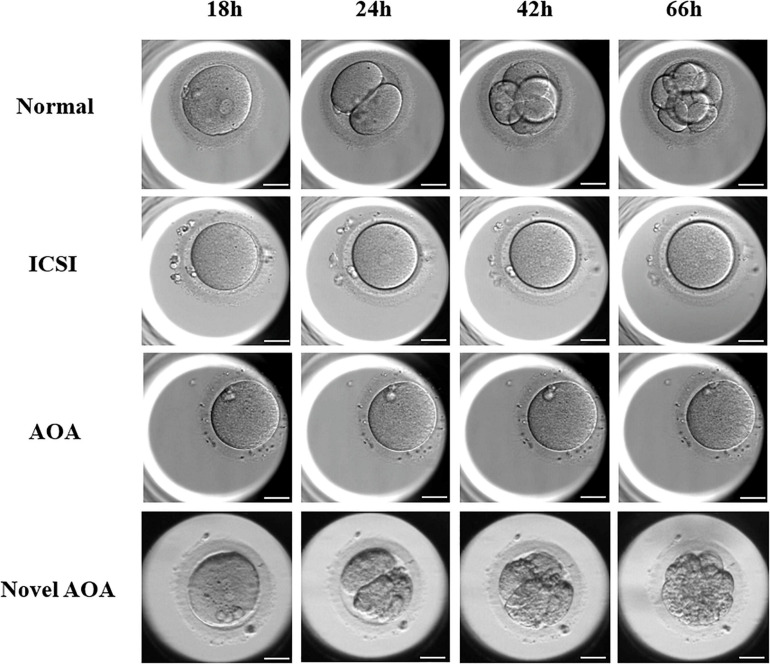
The exhibition of embryos at the different stages using various techniques. Images of embryos at 18, 24, 42, and 66 h after insemination were presented. In the ICSI group and the conventional ICSI-AOA group, PN and cleavage cannot be observed in those oocytes, whereas, the oocytes treated by novel AOA can form pronuclei and develop to cleavage stage embryos normally. Scale bar = 50 μm.

Time-lapse systems were used to monitor the development of embryos from insemination to blastocyst formation. Images of embryos at 18, 24, 42, and 66 h after insemination were captured and collected, and these time points corresponded to the PN stage, 2-Cell stage, 4-Cell stage, and 8-Cell stage of a normal embryonic development, respectively (Figure 2). In the ICSI group and the conventional ICSI-AOA group, PN and cleavage cannot be observed in those unfertilized oocytes, and those successfully fertilized oocytes were not able to develop into good or fair embryos. Moreover, in Case 6, there was no PN exhibited and further embryo development, although the exclusion of second polar can be observed in most of the oocytes. However, after novel AOA treatment, oocytes could be well fertilized and embryos were able to develop into 8-Cell stage embryos or even blastocysts with a good morphology. This indicated that the application of the novel AOA treatment was able to ensure a successful fertilization for couples suffering from TFF after ICSI.

### Aneuploidy Analysis of Embryos Developed From Novel AOA

**FIGURE 3:**
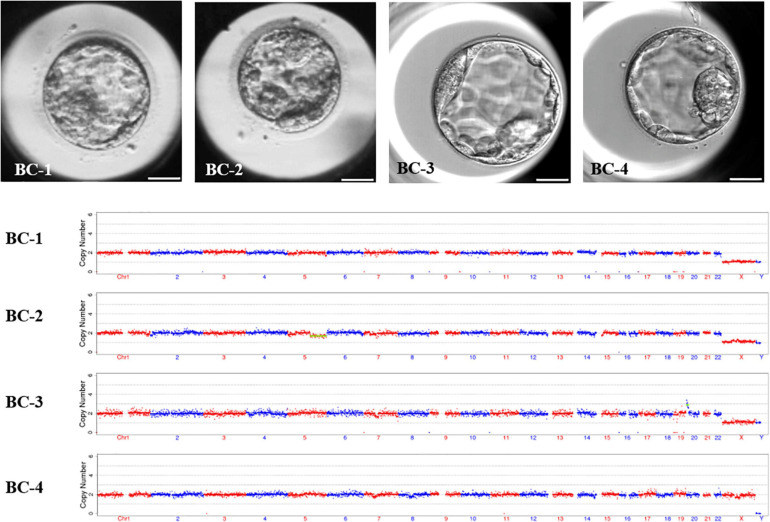
The morphology and karyotype profile of blastocysts resulting from novel ICSI-AOA treatment. In order to detect the aneuploidy of the formed embryos, four blastocysts were selected to analyze the chromosome ploidy by whole-embryos aneuploidy analysis. The karyotypes of these four embryos were 46, XY; 46, XY, -5q (q21.1→qter, ∼76M, ×1, mos, ∼30%); 46, XY, + 20p (pter→p12.2, ∼11.9M, ×3); and 46, XX; respectively. Scale bar = 50 μm.

Novel AOA not only rescues fertilization failure to these special patients, but also, the fertilized oocytes could normally cleave and even develop to good quality blastocysts in the extended culture. In order to detect the aneuploidy of the formed embryos, four blastocysts were selected to analyze the chromosome ploidy by whole-embryo aneuploidy analysis. The morphological scores of the blastocysts at day 5 were 3BC, late stage (stage 2), 4BB, and 4AC, respectively. The results showed that two out of four were normal in terms of the karyotype: one is mosaic and one is abnormal. The detailed karyotypes of these four embryos turned out to be 46, XY; 46, XY,-5q (q21.1→qter, ∼76M, ×1, mos, ∼30%); 46, XY, + 20p (pter→p12.2, ∼11.9M, ×3); and 46, XX; respectively (Figure 3). It was clear that the embryos receiving the novel AOA treatment had the potential to develop into blastocysts with normal karyotypes.

## Discussion

In this study, we presented a novel AOA method applied in six couples suffering from a failed fertilization after previous ICSI and even with conventional AOA and, explored its possible mechanism. Our data showed that CHX combined with ionomycin can effectively improve the fertilization rates and promote a normal embryonic development for couples who experienced repeated ICSI failure.

The pregnancies after ICSI were first reported in the early 1990s, and for decades, ICSI has been the most effective and successful method to treat male factor infertility like obstructive azoospermia (Rubino et al., 2016). Despite the high success rate of ICSI, complete fertilization failures still occur in 1–3% of the total ICSI cycles (Flaherty et al., 1998). Gamete immaturity, abnormal morphology, or inherited genetic defects may account for the failed fertilization (Nasr-Esfahani et al., 2010), and failure of oocyte activation may be the main cause of TFF (Tesarik and Sousa, 1995). In recent years, AOA was advocated to deal with fertilization failure after ICSI, and plenty of studies have shown that the majority of couples experiencing ICSI failure can benefit from AOA (Montag et al., 2012; Sfontouris et al., 2015; Fawzy et al., 2018). Ca^2+^ ionophore is still one of the most popular and effective AOA methods worldwide (Swann, 2018). However, we found that there were still a few couples suffering from fertilization failure even after conventional AOA in our center. Similarly, Yoon also reported 13 patients who experienced fertilization failure even after conventional AOA in 185 patients (Yoon et al., 2013). Conventional AOA based on Ca^2+^ oscillation, such as A23187, is not always effective and beneficial to overcome fertilization failure in all patients (Ferrer-Buitrago et al., 2018; Bonte et al., 2019). Therefore, we hypothesized that Ca^2+^ signaling inactivation could not account for all the patients with TFF, while there may be some other active pathway involved in oocyte fertilization or there may be an abnormal interruption in the cascade downstream activation pathway of Ca^2+^ signaling.

Sperm-derived PLCζ, which participates in the production of Ca^2+^ releasing messenger InsP3, is universally considered as the initiator of Ca^2+^ oscillations at fertilization (Sanders et al., 2018). Some studies have provided evidences that knockout of PLCζ failed to trigger Ca^2+^ oscillations in oocytes, resulting in subfertile male mice (Hachem et al., 2017), and that human recombinant PLCζ protein can elicit Ca^2+^ oscillations after microinjection into mouse oocytes (Kashir et al., 2011). Consequently, PLCζ assays for diagnosis and human recombinant PLCζ for supplement of therapy have gained much attention (Amdani et al., 2016). Significant evidences have indicated that defects in the expression of testis-specific PLCζ correlate with a low success or fertilization failure after ICSI, and abnormal PLCζ1 is considered as one of the possible mechanisms responsible for TFF (Nomikos et al., 2017). In the current study, the immunofluorescence test showed that the expressions of PLCζ were without exceptions in the control group and sperm-related and oocyte-related AOA failure groups. Based on the results obtained for Case 2, the presence of PLCζ1 could indicate that aberrant Ca^2+^ signaling did not take place, and therefore, traditional A23187-based ICSI-AOA did not alleviate fertilization failure.

Due to the significant role of protein kinases and phosphatases in the fertilization process (Adhikari and Liu, 2014; Tosti and Ménézo, 2016), it was available to combine Ca^2+^ ionophores with a protein synthesis inhibitor for parthenogenetic oocyte activation (Naruse et al., 2007; Suvá et al., 2019). CHX, a non-specific protein synthesis inhibitor, can inhibit the synthesis of cyclin B, which inactivates MPF and finally results in the relief of the meiosis arrest (Mori et al., 2008). Although our data have suggested the effectiveness of the novel AOA method, due to the non-specificity of CHX, the exact interaction sites cannot be determined and whether the expression and modification of other proteins will be influenced is not yet clear. Besides, due to the uncertainty of the targets, there may be some unknown side effect of CHX on the embryos, therefore, in the future, a transcriptomic or proteomic analysis would be of value in these cases. In the current study, the chromosome ploidy of four blastocysts developed from the novel AOA method were analyzed by whole-embryo aneuploidy analysis, and three of them were identified to be XY karyotype. It indicated that spermatozoa had also participated in this process and contributed to the formation of the PN and zygote, despite the fact that this combined treatment was originally applied to mammalian parthenogenetic oocyte activation (Kashir et al., 2013).

Although the application of AOA is able to ensure a successful ART for many couples suffering from TFF after ICSI, in part, AOA operation is not a routine option for the majority of IVF centers yet because of the concerns about safety and efficacy. Some studies suggested that AOA may increase the risks of birth defects (Vanden Meerschaut et al., 2014a; Mateizel et al., 2018), while some other studies have found no statistical differences between AOA and non-AOA neonates in terms of gestational duration, gender, birth defects, or birth weight (Deemeh et al., 2015; Miller et al., 2016), so did a meta-analysis performed by our research team, in which five studies were included and the results indicated that ICSI-AOA represented no significant difference in the prevalence of major birth defects or types of birth defects compared with the conventional ICSI (Long et al., 2020). Here, for the first time, we introduced CHX as a promising and effective human oocyte activation agent and provide some preclinical evaluation. Our results demonstrated that CHX-based AOA can improve the fertilization rate and obtain normal embryos. However, CHX, as a non-specific protein inhibitor, may affect other regulatory proteins involved in DNA synthesis and replication. The safety of this agent needs to be further discussed in future researches and clinical practices.

However, there were still several limitations in this study. Firstly, the sample size was limited and it was a single-center study. A large multiple-center study involving more TFF cases is needed in the future to reinforce the existing results and conclusion. Secondly, the exact mechanism of human fertilization failure still remains unknown. Although a rapid rise of fertilization rate can be observed in novel AOA groups, we still cannot confirm the certain interaction sites of our agents, especially CHX. Due to the indeterminacy of the mechanism, further supports are needed in future researches. Moreover, fertilization failure still occurred in one case in our study, which indicates that there are still some situations that this novel AOA method is invalid. Finally, the safety of the AOA agents needs to be further discussed, and the risk of birth defects in children conceived by ICSI-AOA remains ambiguous. More cases and data are needed until its safety is thoroughly verified.

## Conclusion

In summary, we found that CHX combined with ionomycin was able to effectively improve the fertilization rate in the majority of patients suffering from TFF. Remarkably, despite that the certain mechanism of TFF remains unclear, our data showed that this novel AOA had a potential therapeutic effect on those couples experiencing TFF after conventional AOA, which may surmount the severe fertilization deficiencies in patients with repeated low fertilization or TFF.

## Data Availability Statement

The original contributions presented in the study are included in the article/Supplementary Material, further inquiries can be directed to the corresponding authors.

## Ethics Statement

The studies involving human participants were reviewed and approved by the Ethics Committee of Tongji Hospital, Tongji Medical College, Huazhong University of Science and Technology (TJ-IRB20200722). The patients/participants provided their written informed consent to participate in this study.

## Author Contributions

QX, LJ, and ZL conceived the study. MW and LZ wrote the manuscript. MW, CL, JL, and HH performed the experiments. CW, CX, and LY collected and analyzed the data. All authors contributed to the article and approved the submitted version.

## Conflict of Interest

The authors declare that the research was conducted in the absence of any commercial or financial relationships that could be construed as a potential conflict of interest.

## Supplementary Material

Supplementary Table 1 Semen parameters of the participants involved.
