# Corrigendum: A novel assisted oocyte activation method improves fertilization in patients with recurrent fertilization failure

**DOI:** 10.3389/fcell.2023.1176005

**Published:** 2023-03-28

**Authors:** Meng Wang, Lixia Zhu, Chang Liu, Hui He, Cheng Wang, Chenxi Xing, Jinming Liu, Liu Yang, Qingsong Xi, Zhou Li, Lei Jin

**Affiliations:** ^1^ Reproductive Medicine Center, Tongji Hospital, Tongji Medical College, Huazhong University of Science and Technology, Wuhan, China; ^2^ Department of Oncology, Tongji Hospital, Tongji Medical College, Huazhong University of Science and Technology, Wuhan, China

**Keywords:** assisted oocyte activation, total fertilization failure, cycloheximide, ionomycin, intracytoplasmic sperm injection

In the published article, there was an error in the author contribution data on the first page. The corrected author contribution data on the first page appears below.

“The corresponding authors are QX, ZL, and LJ, and contributed equally to this work. MW and LZ share first authorship and also contributed equally.”

The authors apologize for this error and state that this does not change the scientific conclusions of the article in any way. The original article has been updated.

