# Supplementary material for: A Novel Assisted Oocyte Activation Method Improves Fertilization in Patients With Recurrent Fertilization Failure
Source: Front Cell Dev Biol. 2021 Jul 21;9:672081. doi: 10.3389/fcell.2021.672081 (PMC8334862; doi:10.3389/fcell.2021.672081)
Supplement: Supplementary Table 1 — Semen parameters of the participants involved. [file Table_1.docx]

**Supplementary Table 1 Semen parameters of the participants involved.**

|  | **Concentration of (×10^6^/mL)** | **Total motility**  **(%)** | **Normal morphology rate (%)** | **Diagnosis*** |
| --- | --- | --- | --- | --- |
| Case 1 | 6.28 | 27.00 | 8.00 | Oligoasthenotspermia |
| Case 2 | 50.38 | 40.12 | 12.00 | normal |
| Case 3 | 47.70 | 44.40 | 11.00 | normal |
| Case 4 | 47.70 | 71.70 | 1.50 | teratozoospermia |
| Case 5 | 30.10 | 46.70 | 3.82 | teratozoospermia |
| Case 6 | 131.00 | 67.60 | 10.00 | normal |

Note:

*The semen analysis was based on the standard of the fifth edition of the WHO guidelines. A normal semen sample should be equipped with at least a concentration of 15×10^6^/ml, a total motility of 40%, and a normal morphology rate of 4%.
